# Between sound and sleep: a perspective on Sonic Sleep Aids

**DOI:** 10.1093/sleep/zsaf275

**Published:** 2025-09-11

**Authors:** Jessica Vazzaz, Faith Matcham, Marcos Economides, Kate Cavanagh

**Affiliations:** School of Psychology, University of Sussex, East Sussex, United Kingdom; School of Psychology, University of Sussex, East Sussex, United Kingdom; Science Department, Unmind Ltd, London, United Kingdom; School of Psychology, University of Sussex, East Sussex, United Kingdom

**Keywords:** sleep disturbance, smartphone app, digital intervention, sleep aids, digital health, sleep music, mindfulness, sleep hygiene, direct to consumer products

## Abstract

Sleep disturbances are prevalent in the general population, coinciding with a surge in the availability and use of digital sleep aids. Among these, standalone audio-based tools, termed Sonic Sleep Aids (SSA), such as sleep music, ambient sounds, bedtime stories, and sleep skills (e.g. guided meditation, positive psychology techniques), have gained popularity. This perspective piece examines the phenomenon of SSA by discussing the existing evidence and highlighting the different levels of empirical support across SSA types. Music-based relaxation has demonstrated efficacy in improving sleep quality, whereas findings on ambient sounds (e.g. white, pink noise) are inconclusive. Empirical support for narrated content as a sleep aid remains limited. Guided practices like mindfulness and self-compassion show potential, yet further research is needed to support their effectiveness, particularly when limited to bedtime practice. In the broader context, the widespread use of app-based SSA raises questions about their alignment with sleep hygiene recommendations, which typically discourage bedtime screen use. This concern is compounded by a paucity of randomized controlled trials testing their effectiveness against well-matched controls, alongside the risk of increased dependency on technology and altered relationships with rest and introspection. Against these concerns, potential benefits include accessibility and reduced reliance on pharmacological aids. A research agenda is proposed to investigate the efficacy of digitally delivered SSA in naturalistic settings, their mechanisms of action, and their impact across different populations. Understanding these factors is crucial to determine whether SSA serve as beneficial tools or divert individuals from more effective, evidence-based approaches to sleep.

## Teaser

**Statement of Significance** Sleep disturbances affect a substantial portion of the general population, leading many to turn to digital sleep aids, particularly app-based audio tools, in search of relief. Despite their growing popularity and nightly use by millions, there is limited understanding of their effectiveness and how they influence sleep. This perspective piece synthesizes the existing evidence and identifies key knowledge gaps, raising important questions about their broader implications. It considers whether these tools represent a meaningful addition to a toolkit of evidence-based sleep interventions or signal a deeper shift in how individuals relate to rest, technology, and self-regulation. By outlining a future research agenda, this piece highlights the urgent need to evaluate these tools in context and inform their responsible development and use.

## Graphical Abstract

**Graphical Abstract:**
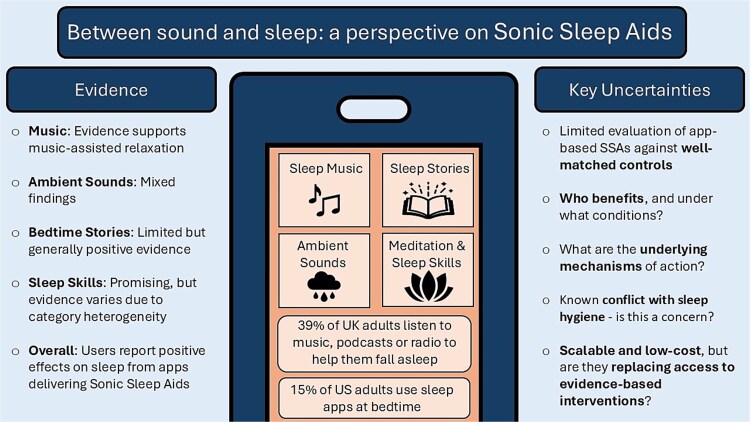

## Introduction

Chronic insomnia affects ~10 per cent of the general population in Western countries, with women 1.5 to 2 times more likely to be affected than men [1]. Its prevalence is also higher among individuals with comorbid physical or mental health conditions [2]. Subclinical sleep disturbances are more widespread, with about one-third of adults reporting weekly sleep problems, and in a 2020 US survey, 14.5 per cent of adults had trouble falling asleep most or every day over the past month [3].

For those who seek medical help, effective treatments are available, including medication [4] and cognitive Behavioral therapy for insomnia (CBTi) [5], the first-line treatment recommended by the National Institute for Health and Care Excellence [6]. However, access to evidence-based care remains limited due to low help-seeking [7] and under-referral. A 2023 analysis of primary care data from three North London boroughs found that only 0.3 per cent of patients identified with insomnia were referred for CBTi [8].

Considering this, it is unsurprising that millions of people who struggle with their sleep worldwide are turning to direct-to-consumer sleep health products. This includes the growth of a large market of “sleep apps,” which may offer a range of features, including digital delivery of treatments and sleep monitoring. Among these are apps that offer Sonic Sleep Aids (SSA), which have seen a marked rise in popularity and are the focus of this perspective piece.

## What are Sonic Sleep Aids

SSA are digital content, delivered via audio files, designed to help people fall asleep and stay asleep. SSA are intended for use during the periliminal sleep period, defined as the time “surrounding the entrance” of sleep. This represents time preparing for sleep, just before attempting to fall asleep and during sleep onset, and, in some cases, continuously throughout the night, including during nighttime awakenings. This content includes colored noises (such as white, pink, and brown noise), soundscapes (such as nature sounds), music, narrated stories (such as audiobooks, podcasts, or bedtime stories), and sleep skills and meditations (such as mindfulness-inspired guided practice or relaxation). SSA can be accessed as needed on a one-off or occasional basis, as a regular intervention program over several nights or weeks or used habitually and indefinitely. SSA can be used either as standalone tools or in combination with other consumer technologies and treatments. For example, some individuals might use SSA independently as a simple, accessible, low-cost aid that requires no daytime commitment, with effects assumed to occur synchronously through passive bedtime listening. Others might use SSA alongside platforms offering CBTi [9], or in conjunction with devices offering hybrid approaches to improve sleep, such as wearable-delivered interventions [10], EEG-enabled headbands that adapt sound content based on real-time feedback [11], music players embedded inside ergonomic pillows [12], or transcranial direct current stimulation [13]. Furthermore, recent advances in artificial intelligence may drive significant innovation, with the promise of increasing personalization of interventions within sleep health products [14], including SSA. Future research should examine how SSA are used and whether these patterns of use may shape engagement and outcomes.

## Popularity, accessibility, and user appeal of SSA

While usage patterns remain unclear, the appeal of SSA is evidenced by their popularity. SSA are widely available on high footfall streaming platforms (e.g. Spotify, YouTube) and through apps focused specifically on sleep (e.g. BetterSleep, Calm) or more broadly on mental well-being (e.g. Headspace, Unmind). While it is difficult to estimate how many people regularly or occasionally use SSA, over 6 million people follow Spotify’s most popular sleep playlist [15], and various soundscapes, stories, and sleepcasts across multiple platforms have attracted anywhere from tens of thousands to over a million followers, downloads, or listens. Nine out of the top 50 health and fitness podcasts on Spotify for the UK are “sleepcasts,” with a podcast dedicated entirely to sleep meditation and hypnosis ranked 7th, and a sleep story podcast in 14th position [16]. The most successful app offering this content, Calm, reported over 150 million downloads worldwide [17]. Thirty nine per cent UK adults report listening to music/podcasts/radio at least occasionally to get to sleep [18], and 15 per cent of US individuals use a sleep app as part of their bedtime routine [19], highlighting the growing engagement with sleep-related content.

These resources clearly have widespread appeal; they are heavily marketed, easily accessible, affordable, and promise to help users “Say goodnight to racing thoughts and drift off to sleep” [20], “help you relax and fall asleep faster” [21], “fall asleep faster, wake up energized” [22], and “Fall Asleep (and stay asleep) naturally and peacefully” [23]. Various healthcare authorities endorse their use. For instance, in England, the National Health Service (NHS) recommends “beditation” noting that “...listening to soft music, a podcast, or sleep meditation can help if you have trouble sleeping” [24].

So, what is the evidence behind these interventions? How do they work, and what outcomes can they deliver, for whom and under what circumstances? This article aims to answer these questions by examining the evidence base for the effectiveness of four popular categories of SSA: colored noises, music-based SSA, narrated stories, and sleep skills and meditation-based SSA. The report will conclude with practical recommendations for further research on SSA, highlighting gaps in the literature and suggesting potential directions for future studies.

## Toward a conceptual framework of the use of SSA

The idea of using sound to alter behavioral or affective states is not unique to periliminal SSA. The increased accessibility of technologies, such as smartphones and noise-canceling headphones, now allows people to craft and control their aural environment to suit their specific needs. This phenomenon, termed “sonic self-control” refers to an individual’s intention to regulate the inflow of sound and information, thereby managing the impact of the environment on the self [25]. Within the context of sleep, several authors have presented hypotheses about how different genres of SSA might be used to positively impact sleep. While the action of individual SSA may vary, several shared underlying mechanisms of action are proposed, including masking possibly sleep-disruptive environmental noises, decreasing pre-sleep arousal by fostering relaxation, distracting from internal or external stimuli, or facilitating other forms of cognitive emotion regulation. These include both shifts toward more positive pre-sleep cognitions and emotions through enjoyment of the SSA [26], and via direct guidance toward eliciting wellbeing and positive emotions such as gratefulness [27] and self-compassion [28], as practiced in some sleep skills and meditation. These could work alone or in conjunction with other underlying mechanisms that might reduce pre-sleep negative cognitive and emotional processes, including fostering acceptance and improving attentional control [29], reducing rumination [30], and repetitive negative thinking [31]. More generally, SSA might operate through a mechanism of harm minimization, by offering a less detrimental way of engaging with phones at bedtime. Given that many individuals use their phones in bed regardless of sleep hygiene recommendations, bedtime technology use has been proposed as a potential emotion regulation strategy or as a time filler [32], regardless of whether these behaviors are conducive to sleep. While bedtime phone use is generally associated with poorer sleep outcomes, different types of use appear to have varying effects [33], supporting the idea that SSA may represent a comparatively less harmful alternative to more stimulating behaviors such as scrolling social media or checking work emails. SSA might also form part of a consistent bedtime routine or ritual or provide a cue to sleep [34]. Finally, given the well-established placebo effect in perceived sleep outcomes [35], SSA effectiveness may partly stem from expectations [26] shaped by cultural norms, research processes, or marketing claims. While this conceptual framework offers a multifaceted rationale for the application of SSA, there is a paucity of evidence directly testing these underlying mechanisms of action, and more research is needed.

## Evidence on the effectiveness of SSA categories on sleep outcomes

Having outlined the theoretical frameworks underpinning proposed mechanisms of action, the following section examines the existing evidence supporting the effectiveness of different categories of SSA in improving sleep outcomes. A summary of relevant research studies can be found in Table 1.

**Table 1:** Overview of studies on bedtime and night-time use of sonic sleep aids (SSA)

Source	Study design	Population (*n* =) and settings	SSA characteristics	Control group	Study duration	Sleep measures	Results summaries
Colored noise and nature sounds							
Riedy et al. (2020) [37]	Systematic review	Mixed (*k* = 25, *n* = 1328)	White noise (*n* = 7), Broadband noise (*n* = 5), air purifier/fan/other motor noise (*n* = 5), variable sounds (*n* = 3), pink noise (*n* = 2), others (*n* = 3)	Various	Various	Subjective: PSQI (*n* = 4), RCSQ (*n* = 2), PROMIS SD (some domains) (*n* = 1), SSA (*n* = 2), GSDQ (*n* = 1), SQVA (*n* = 4), KGSS (*n* = 1), SMSQ (*n* = 1), Ad hoc sleep questionnaire (*n* = 6) Objective: PSG or EGG (*n* = 11), actigraphy (*n* = 2), and observation (*n* = 2).	Perceived Sleep Quality: varied PSG: varied Actigraphy: varied Overall: high heterogeneity of studies, quality of evidence is very low
Capezuti et al. (2022) [36]	Systematic review	Mixed (*k* = 43, *n* = 1103)	White noise (*n* = 18), pink noise (*n* = 10), white noise vs. pink noise (*n* = 1), Multiaudio (*n* = 6)	Various	Various	Subjective: PSQI (*n* = 4), RCSQ (*n* = 4), VSHS (*n* = 1), PROMIS (*n* = 1), ISI (*n* = 1), SSS (*n* = 1) Objective: PSG (*n* = 16), actigraphy (*n* = 5)	Perceived sleep quality: varied results (*n* = 9 positive) PSG: varied Actigraphy: varied Risk of bias: white noise > pink noise > multiaudio (lowest)
Music							
Chang et al. (2012) [38]	RCT	Adults with insomnia (*n* = 50), sleep lab settings	Participant-selected or researcher-selected music (45 min), delivered via CD	None	4 days	Subjective: perceived sleep quality (ad hoc morning questionnaire) Objective: clinical sleep measures (PSG)	Ad hoc questionnaire: significantly higher rested ratings for music; no significant group differences in self-reported total sleep time, sleep onset latency, or number of awakenings PSG: stage 2 sleep significantly shorter and REM sleep significantly longer in music group; no significant effects on other parameters
Deshmukh et al. (2009) [39]	RCT	Adults (*n* = 50) with MDD and sleep disturbances, home settings	Researchers-selected music (45 min), delivered via cassettes	Benzodiazepines medication (mean dose of Diazepam = 7 mg, Chlordiazepoxide = 10 mg)	4 weeks	Subjective: perceived sleep quality (PSQI)	PSQI: no significant differences between music and medication, except at final visit (day 45), where music was significantly better than medication.
Su et al. (2013) [42]	RCT	Inpatients (*n* = 28), in ICU	Music (45 min), delivered via CD	Care as usual	1 night	Subjective: perceived sleep quality (VHS) Objective: clinical sleep measures (PSG)	VHS: significant reduction for music PSG: significantly shorter stage N2 sleep and significantly longer stage N3 sleep, no significant differences in any other measure
Yan et al. (2024) [44]	RCT	University students (*n* = 75) with sleep issues, in dormitory settings	Music (30 min), delivered synchronously via online conferencing software + self-guided three deep breaths and a sleep mantra	Self-guided three deep breath and sleep mantra 30 min before sleep on two consecutive weeknights	5 weeks	Subjective: perceived sleep quality (PSQI), insomnia symptoms (ISI)	PSQI: significant reduction for music vs. control ISI: significant reduction for music vs. control
Lai and Good (2006) [41]	Repeated measures RCT	Older adults (*n* = 60), home settings	Participant-selected sedative music played at bedtime (45 min), delivered via audiotape	None, instructed not to listen to music at bedtime	3 weeks	Subjective: perceived sleep quality (PSQI)	PSQI: significant reduction for music vs. control
Hernandez-Ruiz (2005) [40]	Pre- and post-test design with control and experimental groups	Women (*n* = 28) survivors of domestic abuse, residing in a shelter	Participant-selected music + PMR guidance (20 min), delivered via CD	Silence	1 night	Subjective: perceived sleep quality (PSQI)	PSQI: significant reduction for music vs. control
de Niet et al. (2010) [43]	Quasi-experimental pretest–posttest design with a comparison group	Inpatients with severe mental health conditions (*n* = 54) in a psychiatric hospital	Soothing music, delivered via MP3 player + sleep hygiene education	Care as usual	2 weeks	Subjective: perceived sleep quality (RCSQ subscale)	RCQS sleep quality subscale: significant positive effect for music RCQS Total: no significant effect
Sleep skills and meditation							
Butz and Stahlberg. (2018) [28]	Factorial between-participants	University students (*n* = 143), in home settings	Self-compassion Meditation (20 min) delivered via audio file sent by email OR written task (20 min)	3-min personal problem reflection. Writing task: 20 minu without any instructions	1 night	Subjective: perceived sleep quality (JSS)	JSS: No overall group difference; planned contrast showed higher sleep quality in intervention. No difference between meditation and writing tasks
Multiple SSAs delivered							
Harmat et al. (2008) [45]	RCT	University students (*n* = 94) with subclinical sleep disturbances, in home settings	Music vs. audiobook (30 min), delivered via CD	No Intervention, asked not to use music, and audiobooks at bedtime	3 weeks	Subjective: perceived sleep quality (PSQI)	PSQI: significant effect of music, no significant effect for audiobook Music > audiobook
Jespersen et al. (2019) [46]	RCT	Adults with insomnia disorder (*n* = 57), mixed settings (home and sleep lab)	Music vs. audiobook (used as control) (30 min minimum), delivered via an audio player designed to be used in bed with an inbuilt fade function	WL	3 weeks	Subjective: perceived sleep quality (PSQI), insomnia symptoms (ISI) Objective: PSG, actigraphy	PSQI: no significant group x time effects ISI: no significant group x time effects PSG: no significant group x time effects Music > audiobook
Economides et al. (2023) [53]	Pilot RCT	Working adults with self-reported sleep disturbances (*n* = 300), home settings	Nightwaves (music, colored noise, soundscapes, multiaudio tracks) vs. sleep tales, delivered via a commercial app	WL	4 weeks	Subjective: perceived sleep disturbances (PROMIS SD-SF), sleep-related impairment (PROMIS SRI-SF)	PROMIS SD-SF: significant large effects in favor of both interventions compared to WL PROMIS SRI-SF: significant large effects in favor of both interventions compared to WL Nightwaves = sleep tales
Kirk et al. (2022) [47]	Random crossover design	Adults without sleep disturbances and naïve meditators (*n* = 40), home settings	Sleep Music (45 min) vs. Sleepcasts (45 min) (narrated content) vs. guided mindfulness (15 min minimum), delivered via a music app developed for research purposes	No-intervention baseline (week 1)	Each intervention condition lasted 1 week, with a 1-week washout period between conditions	Subjective: perceived sleep quality (PSQI) Objective: actigraphy	PSQI: significant positive effects for mindfulness only compared to baseline week Actigraphy: significant improvement in sleep efficiency for all conditions compared to baseline week Mindfulness > music/sleepcasts
Mermer and Arslan (2024) [50]	Quasi-experimental pretest–posttest design with a comparison group	Inpatients (*n* = 56), in ICU	Audiobook (mixed with music) (30 min), delivered via audio player	Care as usual	1 night	Subjective: perceived sleep quality (RCSQ)	RCSQ: significant group x time interaction in favor of intervention

Summary of studies examining sonic sleep aids delivered at bedtime and/or during the night. The table presents study design, population and setting, intervention features, control group, study duration, sleep-related outcomes (subjective and objective), and a summary of results. Abbreviations: EEG, electroencephalogram; GSDQ, General Sleep Disturbance Questionnaire; ICU, intensive care unit; ISI, Insomnia Severity Index; JSS, Jenkins Sleep Scale; KGSS, Kwansei Gakuin Sleepiness Scale; MDD, major depressive disorder; PMR, progressive muscle relaxation; PROMIS, Patient-Reported Outcomes Measurement Information System survey; PROMIS SD-SF, Patient-Reported Outcomes Measurement Information System Sleep Disturbance-Short Form; PROMIS SRI-SF, Patient-Reported Outcomes Measurement Information System Sleep-Related Impairment-Short Form; PSG, polysomnography; PSQI, Pittsburgh Sleep Quality Index; RCT, randomized controlled trial, RCSQ, Richards-Campbell Sleep Questionnaire; SMSQ, St. Mary Sleep Questionnaire; SQVA, Sleep Quality Visual Analog scale; SSS, Stanford Sleepiness Scale; VSHS, Verran and Snyder-Halpern Sleep Scale; WL, wait list; >, more effective than; <, less effective than; =, comparable effects.

## Colored noise

A common SSA often used specifically to mask environmental noise is colored noise, with white noise machines being popular and widely available as physical devices as well as digital apps. Despite their widespread use, evidence of their effectiveness remains equivocal. Two independent systematic reviews on colored noise, primarily examining studies on white and pink noise, found no consistent evidence of their effectiveness in improving sleep outcomes [36, 37]. Both reviews identified key methodological limitations, including low overall evidence quality and high risk of bias due to small sample sizes, multi-component interventions, alternative-treatment control groups, and substantial heterogeneity in populations, settings, and interventions. The most consistent improvements in sleep quality were reported in hospital-based studies [36], typically involving participants without primary sleep complaints but in sleep-disruptive environments, where colored noise may mask external disturbances. In contrast, home-based studies, where results are very inconsistent, often recruited individuals with self-reported sleep complaints, though rarely linked specifically to noise [36, 37]. A major limitation of these studies is the lack of assessment or reporting of participants’ auditory environments and the specific properties of the colored noise (e.g. frequency, intensity), making it unclear whether differences in outcomes reflect the intervention itself or its interaction with the surrounding aural environment. Future research should address these gaps with larger, higher-quality randomized controlled trials (RCTs) and careful consideration of environmental and sound-specific variables. Establishing the effects of individual noise types from pragmatic studies of widely available SSA is challenging, as they are often combined with other audio elements, such as nature sounds or music, in multi-audio tracks.

## Music

Research consistently indicates that listening to music during the periliminal sleep period can improve perceived sleep quality in a variety of settings and populations [38–45]. However, one study in individuals with insomnia found only a trend toward improvement [46], and another in healthy adults with no sleep complaints reported no effects [47], with limited capacity for improvement likely due to people being satisfied with their sleep already. Where described, the audio features of music used in research settings align with analyses of Spotify “sleep playlists,” despite substantial variation being reported [48]. This is consistent with evidence suggesting that music genre choice reflects personal preference [49] and that there are no significant differences in outcomes between participant-selected and researcher-selected music [38]. One home-based study found music comparable to benzodiazepines, with greater reductions in perceived sleep disturbance by day 45 in the music group, possibly due to medication tolerance [39]. This suggests music may be a more sustainable alternative, though the literature’s short follow-up periods mean it is unclear if the positive effects of music are sustained over time. Objective sleep outcomes are less frequently studied and show fewer promising results. Two polysomnography studies found reductions in stage 2 sleep but no significant impact on total sleep time, sleep onset latency (SOL), or sleep efficiency (SE) [38, 42], while one actigraphy study in healthy adults reported improved SE but no other effects [47]. In synthesis, music appears to be a safe and effective way to improve perceived sleep quality but may have limited influence on objective sleep metrics. Future research should prioritize large-scale studies conducted in real-world contexts. Integrating self-selected music from sleep apps or streaming platforms with sleep data from wearable devices could generate ecologically valid insights into how music is used as a sleep aid in everyday life, and whether improvements are sustained over time or subject to a plateau effect.

## Narrated stories

Despite their widespread use, much less research on narrated stories at bedtime is available. While there is some support for the use of audio books in improving sleep and some vital functions, in patients in an intensive care unit [50], elsewhere audiobook exposure (30–45 min per night for 3 weeks) was not found effective compared to a waitlist control on insomnia symptoms or objective sleep parameters in participants with insomnia [46] nor students with self-reported sleep complaints [45]. However, a key limitation in the current research on audiobooks as SSA is the lack of detailed reporting on the characteristics of the audio content, which may influence intervention effectiveness. Crucial information, such as the narrator’s voice properties (e.g. gender, accent, timbre, pitch, and intensity), speech rate, and the type of language used, is often omitted. Moreover, it is unclear whether participants were given any choice or control over these features, or whether they were exposed to a single narrator or style. This is important, as personal preference and need for variety may play a role in the enjoyment of the content, as shown for other types of SSA [49, 51]. This lack of variety and personalization is a significant distinction between the audiobooks used in this research setting and sleep stories offered in “sleep apps,” which typically provide a wide range of content tailored to different preferences. Moreover, unlike audiobooks that may be repurposed as sleep aids, app-based bedtime stories for adults are specifically crafted to promote sleep, featuring unique qualities. For example, these stories often incorporate vivid descriptions of calming environments or dreamy landscapes, supplemented by sound effects like nature sounds or relaxing music and relaxation prompts [52]. Cross-sectional evidence indicates that sleep stories are very popular with sleep app users, who also report positive impacts on their sleep [52]. A pilot study comparing sleep sounds (e.g. music and colored noise) and bedtime stories delivered through a mental health app (unmind) to adults reporting sleep disturbances found that both interventions, compared to waitlist controls, significantly improved self-reported sleep disturbances, sleep-related impairment, and mental health and well-being outcomes [53]. These results were not fully replicated in a within-subject crossover study investigating the effects of SSA on subjective and physiological sleep measures in individuals without sleep disturbances. No significant improvements in self-reported sleep quality, compared to baseline, were observed for either music or “sleepcasts” (Headspace-narrated stories). The discrepancy in findings compared to previous research [52, 53] could be attributed to differences in baseline sleep disturbances. This suggests that individuals with sleep disturbances may benefit more from SSA, which is supported by research showing that those with more severe disturbances tend to experience greater benefits [52]. These findings suggest that the efficacy of narrated stories may depend on their specific type, baseline levels of sleep disturbances, and context of use, presenting a complex picture and highlighting the need for further research.

## Sleep skills and meditation

Another popular type of SSA is sleep skills and meditation, which includes guided practices such as relaxation techniques, mindfulness, breathwork, and self-compassion. Evidence suggests that both daytime relaxation [54] and mindfulness meditation training [29] can improve sleep outcomes, and self-help, app-based meditation content has also shown some promise [55–58]. But while the evidence supports daytime practice of these skills, there is ongoing debate over the suitability of mindfulness as a direct sleep aid at bedtime, particularly for individuals with insomnia or novice meditators [59]. Despite this, sleep meditations are highly popular, and qualitative studies indicate that people actively use these techniques to help them fall asleep [51, 60, 61] regardless of the limited evidence base.

Some support for app-delivered mindfulness at bedtime is reported in a crossover study by Kirk and colleagues (2022). Here, guided mindfulness significantly improved various sleep-related outcomes in adults without sleep disturbances. Notably, only mindfulness, unlike sleep music or narrated stories, led to significant improvements in self-reported sleep quality and perceived stress. While all conditions enhanced actigraphy-measured SE, the mindfulness condition yielded the greatest improvements. Additionally, although each condition increased heart rate variability (HRV) during the pre-sleep period, only mindfulness showed a significant HRV increase during sleep, suggesting potential added benefits from this type of SSA [47]. It would be valuable to replicate this study in individuals with sleep disturbances to determine whether similar benefits extend to this population.

Evidence for other types of sleep meditation content is even more limited. Some research supports the use of slow, paced breathing at bedtime to reduce SOL, decrease awakenings, and improve SE [62], while one study suggests that a self-compassion exercise may improve sleep quality compared to a control [30]. However, these findings come with significant limitations, including small sample sizes [62] and reliance on single-session interventions [30], underscoring the need for further research. Evidence supporting bedtime meditation as a standalone intervention to aid SOL specifically is scarce, partly because practice frequency is often prescribed (e.g. daily), but the timing of practice is rarely recorded or reported. Higher-quality, specific RCTs are needed to draw firm conclusions about whether practicing sleep skills and meditations exclusively at bedtime is effective in improving perceived sleep quality, and whether certain techniques confer greater benefits than others. Nonetheless, some of these techniques are endorsed by the NHS [63] and are widely accessible through sleep apps.

## Synthesis of research evidence on SSA

In summary, evidence for colored noise is mixed [36, 37], whereas music interventions show more consistent benefits across settings, with reliable improvements in perceived sleep quality [38–45]. This may reflect differences in study quality or indicate that music has broader appeal or greater tolerability. Additionally, there is a lack of empirical evidence regarding the use of narrated content as sleep aids [53], as it remains unclear which specific characteristics may influence their effectiveness. Guided meditation practices (based on mindfulness, breathwork, and self-compassion practices) each show promise [30, 47, 62], but further evidence is needed, especially given the variety of techniques within this category and the differing strengths of the supporting evidence. Crucially, it is essential to determine whether app-based delivery of SSA effectively promotes better sleep, for whom, and under what circumstances as results may vary based on factors such as the type of population studies (e.g. individuals with insomnia vs. general sleep complaints), the setting (e.g. home vs. hospital), and the characteristics of the tools used and the outcomes measured. A significant limitation of the current evidence in app-based studies is its reliance on waitlist controls, which may lead to an overestimation of treatment effects influenced by expectation and placebo effects. Future studies should compare the efficacy of these interventions to active control conditions and evidence-based interventions (e.g. app-delivered CBTi) to determine whether the observed effects arise from the sleep-enhancing effects of SSA or simply from engaging with a mental well-being app and dedicating time to one’s health, and how these effects compare to well-established sleep improvement approaches. Finally, research should focus less on individual products (such as specific sleep or mindfulness apps) and more on shared, replicable mechanisms as outcomes [64]. This is particularly relevant for app-based SSA, as much of the research on their effectiveness focuses on individual apps and may not be generalizable beyond the unique user experience features of that platform. Additionally, conflicts of interest must be addressed, given that much of the research is funded or authored by commercial entities that may stand to benefit from favorable results. Independently funded research is vital to ensure unbiased results.

## The regulatory landscape of SSA

As these apps are considered “sleep aids” rather than insomnia treatments, they are not subject to the same strict evaluation requirements as medical devices. This regulatory gap allows companies to make claims about their effectiveness without providing evidence, creating a digital health ecosystem driven more by marketing than science [64]. Without robust evidence, it is difficult to determine whether periliminal SSA are effective sleep aids or merely a popular red herring. While their use may be benign, it is crucial for researchers, practitioners, and the public to be aware of the limitations of the current evidence. Additionally, the widespread promotion and availability of these resources could mislead users into believing they are validated therapeutic tools, free from potential adverse consequences.

## Potential benefits and risks of using SSA

Just because many of these products fall under the unregulated category of “wellbeing” tools does not guarantee they are safe or harmless. Although reports of adverse effects in digital mental health research are rare [65] and no significant adverse effects have been reported for sleep music or bedtime stories [53], potential risks associated with their use remain largely unexplored. These risks may include delays in seeking help, failure to address underlying causes of poor sleep, demoralization (e.g. “If this doesn’t work for me, maybe nothing will”), and disregard of evidence-based approaches for managing significant sleep disturbances. Sleep experts caution against “grabbing at solutions,” instead recommending trusting and expecting sleep to come spontaneously [66] (p. 6). This approach may conflict with the reliance on SSA as a crutch for inducing sleepiness. On the other hand, if SSA prove effective and safe, they could offer a scalable solution for sleep impairment accessible to the general population. Long-term use of SSA may help prevent individuals with sleep disturbances from developing clinical insomnia, thereby improving individual health and reducing the strain on public healthcare services, such as the NHS. Conversely, they may also represent an unnecessary or even counterproductive form of techno-solutionism that disrupts natural sleep processes. Further research is needed to examine these competing hypotheses.

## S‌SA and broader societal trends

Regardless of whether SSA are effective, their widespread use may reflect broader societal trends. It could be indicative of the growing prevalence of stress and mental health issues, both of which are known to affect sleep. Furthermore, it may suggest that technology has increasingly become embedded in daily life, with once-silent moments now filled by “sleep content”—a new form of media that transforms periods of non-consumption into opportunities for entertainment within the so-called attention economy, potentially making it monetizable. This shift may also point to changing relationships with silence and introspection, reflecting a drive to seek constant mental engagement throughout the day. Alternatively, it might signify a broader trend of outsourcing traditionally internal processes, such as emotional regulation and the slowing down of thoughts, to external tools. People have historically shared songs (lullabies) and stories at bedtime, a tradition still preserved for many in childhood, but moving toward technology to fulfill this role may have unintended consequences for intergenerational and interpersonal relationships. Conversely, access to digital sleep aids may offer a less harmful alternative to substances such as alcohol or other sedatives commonly used to promote sleep.

While these societal trends are complex and difficult to study empirically, they should not be overlooked, as each brings its own consequences. Sleep may be the canary in the coal mine, reflecting deeper shifts in human behavior and technology, for better, or for worse.

## Future research agenda

Building on the discussions above, the following research agenda is proposed:

1. Further examination of the effectiveness of SSA in well-designed research studies that reflect the naturalistic use of SSA in the general population, including responsive use in the context of sleep difficulty, as well as longer-term programmatic or habitual use of SSA at bedtime. In particular:
**Population:** Identify for whom these tools are effective. Are they suitable for individuals with subclinical sleep disturbances, those with clinical insomnia, or healthy sleepers experiencing occasional bad nights? Could they serve as sleep enhancers for otherwise healthy individuals?
**Intervention:** Determine whether different categories of SSA are effective and if specific types offer distinct benefits.
**Control:** Evaluate whether their effectiveness holds up against credible digital mental health placebos and existing evidence-based interventions.
**Outcome:** Evaluate their effectiveness in improving sleep perception (e.g. self-reported sleep quality) and/or objective sleep measures (e.g. actigraphy). Incorporate more objective measures, such as time in bed and actions taken prior to bed, to gain a better understanding of SSA and insights into circadian rhythm patterns.
2. Further consideration and exploration of reach, uptake, engagement and attrition of SSA, measured more robustly and in a standardized and objective way to allow for greater understanding of the relationship between SSA engagement and outcomes.
3. Further consideration of the mechanisms of action of SSA. A range of theoretical underpinnings are proposed for the potential effects these popular resources including masking possibly sleep-disruptive environmental noises, fostering relaxation, distracting from internal or external stimuli [26], increasing sleep-conductive pre-sleep cognitions and emotions (through enjoyment [26], gratitude [27], and self-compassion [28]) and reducing sleep-disruptive pre-sleep cognitions (through fostering acceptance, improving attentional control [29], reducing rumination [30], and repetitive negative thinking [31]), providing a conditioned sleep cue [34], and as harm-reduction strategy, by substituting to other phone-based activities that might be more detrimental to sleep (e.g. gaming, social media use). There is a paucity of empirical studies testing these hypotheses, which might help us to develop more effective SSA approaches.
4. To explore moderators of SSA, specifically in relation to predictors of engagement and outcomes so that SSA can be more effectively personalized and targeted at those who are likely to benefit most.
5. Component analysis to identify and compare the active ingredients of SSA so that the content most likely to have positive effects can be promoted.
6. For studies utilizing SSA to actively seek out and report any adverse events or other negative effects of use in order to better inform theory, research, and delivery of SSA, and help to better ensure user safety.
7. Through qualitative approaches, gain a better understanding of the use of SSA in a broader context among adults with self-reported sleep disturbances to shed light on the role of technology in sleep.

To conclude, SSA is a multi-million dollar industry and public health phenomenon that remains unregulated and under-researched. It is essential for sleep scientists to engage with SSA to expand our knowledge base and update sleep guidelines accordingly, as part of a broader effort to prioritize sleep health for all and foster a healthy relationship with technology.

## Data Availability

Data sharing is not applicable to this article as no new data were created or analysed in this study.
